# Taxonomy of Arabian *Temnothorax* Mayr (Formicidae: Myrmicinae) with description of a new species enhanced by x-ray microtomography

**DOI:** 10.1038/s41598-019-47260-y

**Published:** 2019-07-29

**Authors:** Mostafa R. Sharaf, Abdulrahman S. Aldawood, Evan P. Economo, Aijaz Ahmad Wachkoo, Francisco Hita Garcia

**Affiliations:** 1 Department of Plant Protection, College of Food and Agriculture Sciences, King Saud University, Riyadh, Saudi Arabia; 2 Department of Zoology, Govt. Degree College, Shopian, Jammu and Kashmir 192303 India; 3 Biodiversity and Biocomplexity Unit, Okinawa Institute of Science and Technology Graduate University, Onna-son, Okinawa, Japan

**Keywords:** Zoology, Entomology

## Abstract

*Temnothorax elmenshawyi* sp. n., a new ant species from the Asir Mountains of the southwestern region of the Kingdom of Saudi Arabia, is described based on the worker caste. The new species is a member of the *T. exilis* species group and is distinguished from the other species included in this group by the impressed metanotal groove, the short, acute and broadly-based propodeal spines, the finely punctate posterior half of cephalic surface, and absence of a median clypeal carina. Despite extensive collecting by the authors at the type locality, only two specimens are available for description, suggesting that this species may be rare and likely endemic to the Asir Mountains. The species description is complemented by still images of volume renderings of a 3D model and a 3D rotation video of the holotype based on x-ray microtomography (micro-CT), allowing remote in-depth examination of the specimen. The virtual micro-CT data is provided as cybertype dataset and freely available online 10.5061/dryad.4gg39k6, as well as 3D surface model (Sketchfab.com, https://skfb.ly/6HYRz). An updated identification key to the Arabian species is presented.

## Introduction

The ant genus *Temnothorax* Mayr^1^ is one of the largest genera in the Family Formicidae with 450 valid species and subspecies known worldwide from all zoogeographic regions^2–5^. Species of this genus inhabit a wide range of habitats from deserts to rainforests^6^. The taxonomic history of the genus is relatively long and complex including the synonymy with *Leptothorax* Mayr, 1855^2,7^ and subsequent removal from synonymy^8–10^. The complete nomenclatorial history of the genus is available in Bolton^3^. Recently, Prebus^5^ reviewed the evolution, biography, and natural history of the genus postulating a Nearctic origin during the Eocene-Oligocene with a shift to arboreal nesting habits during the Oligocene.

The genus *Temnothorax* is diagnosed for the Arabian Peninsula by the following characters for the worker caste^11^: Antennae 12-segmented with a 3-segmented club; mandibles armed with five teeth; palp formula 5, 3; clypeus broadly inserted posteriorly between frontal lobes; anterior clypeal margin convex in full-face view, not forming an apron over mandibular surface in profile; promesonotal suture absent; propodeum bidentate. Members of the *T. exilis* species group can be characterized by the following characters^12^: colour usually dark brown or black; promesonotal suture distinct; propodeal spines short and acute; petiolar node in profile triangular; body sculpture feeble.

The field of insect taxonomy has advanced at great pace within the last decades through the implementation of new tools and methods, such as DNA barcoding (e.g.^13,14^), molecular phylogenetics (e.g.^15,16^), morphometrics^17^, or integrative approaches combining different lines of evidence^18^. Recently, interactive and three-dimensional (3D) imagery, such as x-ray microtomography (micro-CT), is gaining popularity and momentum within arthropod systematics. Micro-CT is a cutting-edge imaging technology that generates high-resolution, virtual, and interactive 3D reconstructions of whole specimens or particular body parts, thus allowing a maximum of morphological accuracy and fidelity^19–21^. Furthermore, a crucial benefit of applying micro-CT for insect taxonomy is the use of openly available cybertype datasets linked to the original, physical type material^19,22,23^.

Sharaf *et al*.^24^ provide the sole treatment of *Temnothorax* for the Arabian Peninsula and recognize and key out three species: *T. arabicus* Sharaf & Akbar, 2017, *T. liviae* (Agosti & Collingwood, 2011), and *T. megalops* (Hamann & Klemm, 1967). Sharaf *et al*.^24^ described *T. arabicus* from the Asir Mountains, Kingdom of Saudi Arabia (KSA), based on the worker caste and reviewed the available regional records for the genus. In the present work, another new species of the *Temnothorax* is described from the Asir Mountains (KSA) based on the worker caste.

## Results

### Synoptic list of Arabian *Temnothorax* species

*Temnothorax arabicus* Sharaf & Akbar, 2017.

*Temnothorax elmenshawyi* Sharaf, Wachkoo, Hita Garcia **sp. n**.

*Temnothorax liviae* (Agosti & Collingwood, 2011)

*Temnothorax megalops* (Hamann & Klemm, 1967)

### *Temnothorax elmenshawyi* Sharaf, Wachkoo, Hita Garcia sp. n

**Figure 1:**
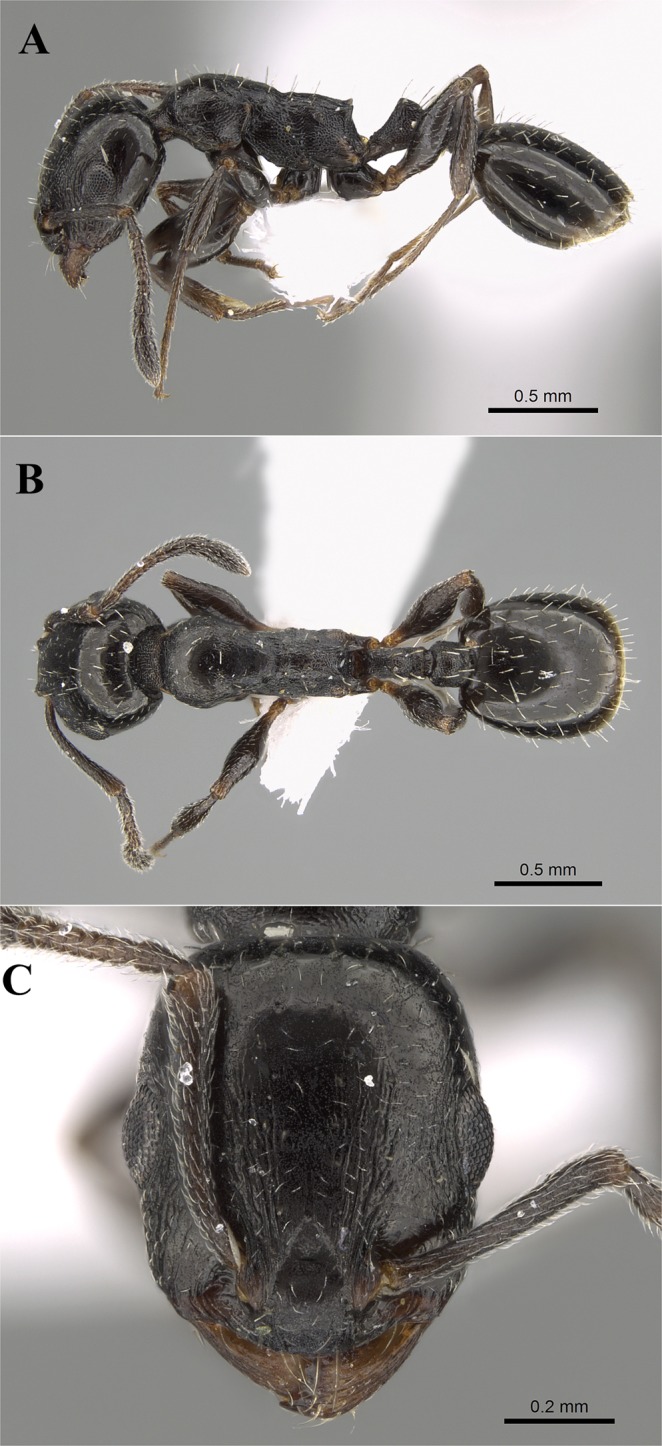
(**A**–**C**) *Temnothorax elmenshawyi* sp. n. holotype worker (CASENT0922350 - photographer: Michele Esposito, AntWeb). (**A**) Body in profile; (**B**) Body in dorsal view; (**C**) Head in full-face view.

**Figure 2:**
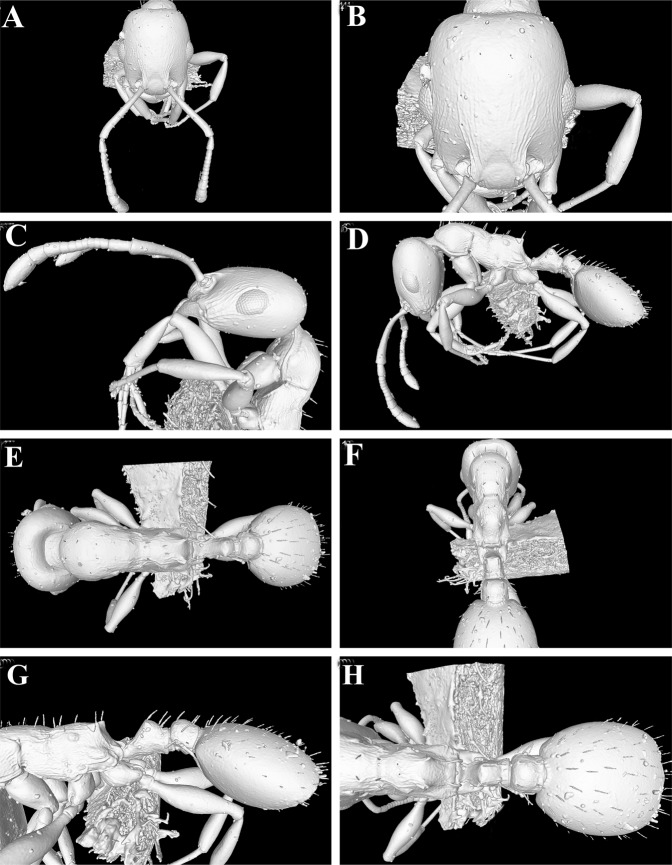
(**A**–**H**) Still images from surface display volume renderings of 3D model of *Temnothorax elmenshawyi* sp. n. holotype worker (CASENT0922350). (**A**) Head (including antennae) in dorsal view; (**B**) Cephalic dorsum in dorsal view; (**C**) Head (including antennae) in profile; (**D**) Body in profile; (**E**) Body in dorsal view; (**F**) Mesosoma and waist segments in posterodorsal view; (**G**) Mesonotum, propodeum, waist segments, and gaster in profile; (**H**) Propodeum, waist segments, and gaster in dorsal view.

**Holotype worker**. Saudi Arabia: Asir Province, Abha, Raydah, 18.201583°N, 42.408933°E, 2578 m., 31.vii.2015, Al Dhafer *et al*., deposited in the King Saud University Museum of Arthropods (CASENT0922350), College of Food and Agriculture Sciences, King Saud University, Riyadh, KSA. **Paratype worker**. One worker with same data as the holotype, also deposited in the King Saud University Museum of Arthropods (CASENT0790240) (Figs 1A–C, 2A–H).

Holotype measurements (paratype in brackets). EL 0.20 (0.17); FRS 0.15 (0.12); HL 0.75 (0.75); HW 0.62 (0.57); IOD 0.52 (0.52); MD 0.25 (0.20); PPH 0.20 (0.17); PPL 0.15 (0.17); PPW 0.20 (0.22); PTH 0.22 (0.22); PTL 0.27 (0.22); PTW; 0.15 (0.12) PW 0.45 (0.40); SL 0.55 (0.45); SPST 0.22 (0.15); WL 0.92 (0.87). Indices. CI 83 (76); DPeI 56 (55); DPpI 133 (129); LPpI 75 (100); OI 32 (30); PeNI 33 (30); PPI 133 (100); PpNI 44 (55); PSLI 29 (20); SI 73 (60).

Cybertype. Volumetric raw data (in DICOM format), 3D rotation video (in.mp4 format, T.*elmenshawyi*_CASENT0790240_video.mp4, see Suppl. material XXX), still images of surface volume rendering, and 3D surface (in PLY format) of the physical paratype (CASENT0790240) in addition to montage photos illustrating head in full-face view, profile and dorsal views of the body. The data is deposited at Dryad^23^ (10.5061/dryad.4gg39k6) and can be freely accessed as virtual representation of the type. In addition to the cybertype data at Dryad, we also provide a freely accessible 3D surface model of the holotype at Sketchfab (https://skfb.ly/6HYRz).

**Diagnosis**. *Temnothorax elmenshawyi* can be distinguished from other members of the species group by the impressed metanotal groove, the short, acute and broadly based propodeal spines, the finely punctate posterior half of cephalic surface, and the absence of median clypeal carina.

### Description

**Head**. In full-face view distinctly longer than broad with nearly straight posterior margin, rounded corners and feebly convex sides; anterior clypeal margin entire and convex; frontal carinae short and distinctly failing to reach anterior margin of eyes in full-face view; mandibles armed with five teeth decreasing in size from apex to base; antennae 12-segmented; scape relatively short (SI 60–73) clearly not reaching posterior margin of head by about length of second funicular segment in full-face view; eyes moderately large (EL 0.29–0.32 × HW, OI 30–32) with about 16 ommatidia in the longest row. **Mesosoma**. Promesonotal suture indistinct; promesonotum flat in profile; metanotal grove distinct; propodeal spines short, acute and broadly based (PSLI 20–29). **Petiole**. In profile without a peduncle; the anterior face forms a shallow concavity anteriorly; anterior face of petiolar node forms a right angle with posterior face; subpetiolar process reduced to a small denticle. **Postpetiole**. In profile globular (LPpI 75–100) and relatively lower than the height of the petiole; in dorsal view trapezoidal broadest anteriorly, 1.2–1.3 broader than long (DPpI 129–133). **Sculpture**. Mandibles longitudinally rugulose; clypeus and cephalic surface behind posterior levels of eyes to posterior margin of head mostly unsculptured medially and shiny, laterally with sparse punctate ground sculpture; cephalic surface starting from posterior margins of clypeus to posterior level of eyes faintly longitudinally irregularly rugulose; dorsal surface of mesosoma densely and finely punctate; lateral sides of mesosoma densely punctate; area between mesopleura and metapleura with distinct longitudinal rugae; promesonotum and mesonotum smooth in dorsal view; propodeum irregularly rugulose; petiole and postpetiole densely and finely punctate; gaster smooth and shining. **Pilosity**. Anterior clypeal margin with six protrusive setae, two short lateral and four central longer ones; clypeus and cephalic surface with appressed scattered pubescence; posterior margin of head with four pairs of erect setae; antennae with abundant appressed pubescence; promesonotum with seven pairs of blunt stiff, short erect setae; mesonotum and propodeum each with two pairs of setae; propodeal spines with one pair of setae; petiole with three pairs of longer setae directed posteriad; postpetiole with five pairs of setae; gaster with scattered blunt setae. **Colour**. Uniformly black, tibiae and tarsi pale brown.

**Queens and Males:** Unknown.

**Etymology**. This new species is named in the honor of the late Egyptian Qur’an reader Mohamed Siddiq El-Menshawy (1920–1969).

**Figure 3:**
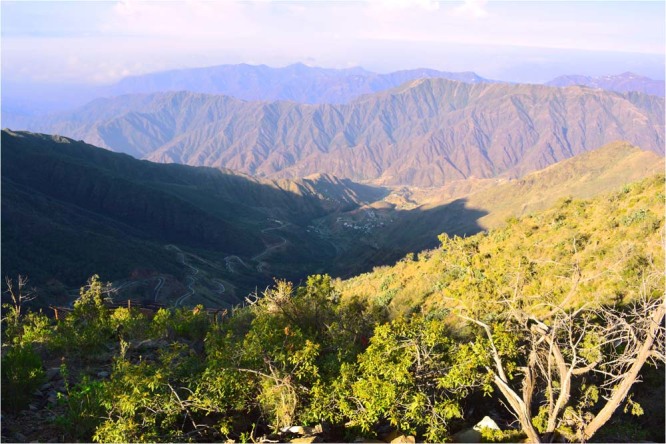
Type locality, Raydah, the Asir Province, Kingdom of Saudi Arabia, (photographer: Ahmed Shams Al ‘Ola).

**Habitat**. The type locality (Raydah) (Fig. 3) is located in the Asir Mountains, 10 km west of the city of Abha and with an estimated area of 9 km^2^ ^16^. This area includes one of the last remnants of dense juniper forests (African pencil cedar, *Juniperus procera* Hochst. ex Endl. (Cupressaceae) remaining on the Arabian Peninsula.

**Biology and Ecology**. Nothing is known of the biology and ecology of the new species.

### Key to the Arabian Temnothorax Mayr (worker caste)

**Figure 4:**
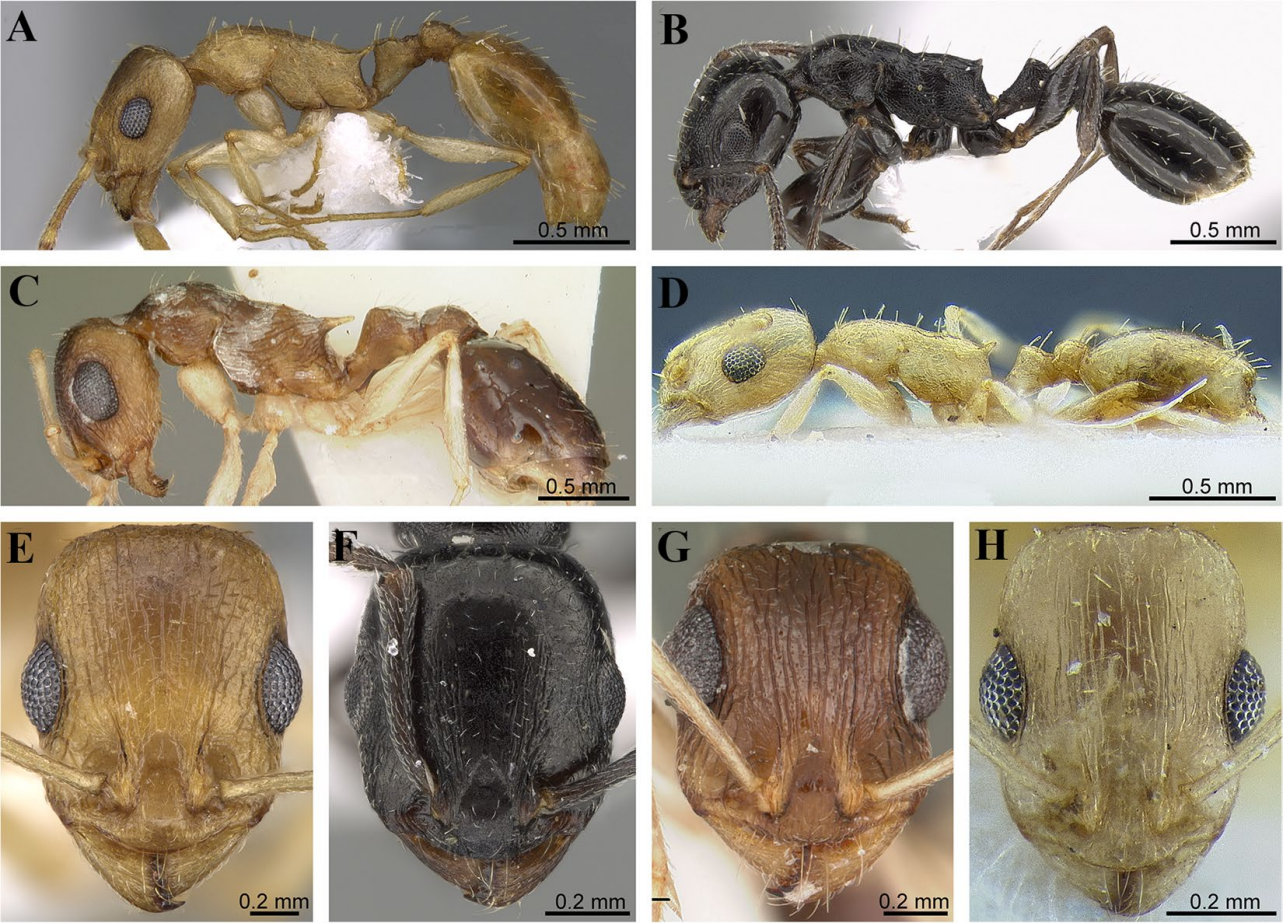
(**A**–**H**) Morphological overview of Arabian *Temnothorax* fauna. Body in profile and head in full face view. (**A**,**E**) *Temnothorax arabicus* Sharaf & Akbar (CASENT0746640 – AntWeb, photographer: Zach Lieberman); (**B**,**F**) *Temnothorax elmenshawyi* sp. n. (CASENT0922350 – AntWeb, photographer: Michele Esposito); (**C**,**G**) *Temnothorax liviae* (Agosti & Collingwood) (CASENT0102700 – AntWeb, photographer: April Nobile); (**D**,**H**) *Temnothorax megalops* (Hamann & Klemm) (CASENT0712601 – AntWeb, photographer: Matthew Prebus).

1. Eyes exceptionally large (OI 46) (Fig. 4C,G)………………………………………..*T. liviae*

- Eyes well developed but significantly smaller (OI 32–37) (Fig. 4A,B,D–F,H)……..2

2. Body colour very dark brown to blackish; cephalic dorsum and sides of head behind posterior eye level mostly unsculptured and shiny with sparse punctate ground sculpture (Fig. 4B,F) ………………………………………………………………………………..…….*T. elmenshawyi*

- Body colour yellowish; most of head with distinct rugose/rugulose sculpture (Fig. 4A,D,E,H)………………………………………………………………………………………………3

3. Frontal carinae well-developed; propodeal spines long and acute; petiole with two pairs of erect setae (Fig. 4A,E)……………………………………………………………………………………*T. arabicus*

- Frontal carinae feeble; propodeal spines short and blunt; petiole with a single pair of erect setae (Fig. 4D,H)…………………………………………………………………………………………………..*T. megalops.*

## Discussion

This new species belongs to the *T. exilis* species group as defined by Cagniant & Espadaler^12^ in the inventory of Moroccan *Temnothorax*, and will not key successfully to any of the Moroccan or African species^4^. It has to be pointed out though, that the *T. exilis* species group is not a monophyletic group based on a recent global phylogeny of the genus^5^, which was already suspected by Cagniant & Espadaler^12^. Without any other existing higher-level classification above species level available for the genus, we prefer to place *T. elmenshawyi* in the *T. exilis* group, at least temporarily.

*Temnothorax elmenshawyi* superficially resembles *T. exilis* (Emery) from Italy but can be readily distinguished by the deeply impressed metanotal groove, the finely punctate posterior half of the cephalic surface, and absence of median clypeal carina. Workers of *T. exilis* have an indistinct metanotal groove, acute propodeal spines, a reticulate-rugulose posterior half of cephalic surface, and a median clypeal carina. With regards to the Arabian *Temnothorax* fauna, *T. elmenshawyi* cannot be confused with any of the Arabian species. It can be immediately separated by the uniformly very dark brown to blackish colour (vs. yellowish in the other three species), moderately large eyes (vs. extremely large eyes in *T. liviae*), and the mostly unsculptured posterior half of the head behind the level of the eyes (vs. conspicuously rugulose/rugose in the other three species).

More than 220 pitfall traps were placed in the Asir Mountains during two years of sampling and only two specimens of the new species were trapped. Undoubtedly, this may reflect the scarcity of this species and indicate a high probability of it being a regional endemic. Due to the extension of the Asir Mountains into Yemen, *T. elmenshawyi* may also occur in that country. It is hoped that future surveys of ants of this region will confirm this distribution, and result in the discovery of additional specimens, especially the male and queen castes.

As discussed in previous studies^19,21^, the use of micro-CT has great advantages for taxonomy. The non-destructive x-ray scan allows the generation of a virtual avatar or cybertype of the physical type material, providing a detailed and almost complete virtual reconstruction of the morphology, thus permitting a high degree of interactive examination for users without access to the physical specimen. Since standard ant taxonomy is still mostly based on the study and comparison of external morphology, virtual study of cybertype datasets can obviate the need to obtain loans from natural history museums, which are often difficult to organize, or to travel to the actual museum collection, which are usually costly and time-intensive. This is of special importance for developing countries where loans are almost never shipped to and travel budgets rather limited. The cybertype provided in this study is the first such virtual 3D dataset for an insect from the Arabian Peninsula or for the ant genus *Temnothorax*.

## Material and Methods

### Measurements and indices

The specimens were measured with Leica M205C stereomicroscope at magnifications of up to 160x. Measurements are expressed in millimeters and following^24–26^.

EL Eye length: maximum diameter of compound eye measured in oblique lateral view.

FRS Distance of the frontal carinae immediately caudal of posterior intersection points between frontal carinae and lamellae dorsal of torulus.

HL Head length: maximum distance from mid-point of anterior clypeal margin to mid-point of posterior margin of head, measured in full-face view.

HW Head width: width of head directly behind eyes, measured in full-face view.

IOcD Inter-ocellar distance: minimum distance between posterior-most pair of ocelli.

IOD Inter-ocular distance: minimum distance between compound eyes, measured in full-face view.

MD Malar distance: minimum distance between anterior margin of compound eye and base of mandible.

PH Petiole height: maximum height of petiole, measured from apex of node to ventral edge of petiole, parallel to anterior margin of petiole.

PL Petiole length: maximum length of petiole node measured in dorsal view from anterior notch close to propodeum to articulation with postpetiole.

PPH Postpetiole height: maximum height of postpetiole measured in lateral view from highest (median) point of node to ventral outline.

PPL Postpetiole length: maximum length of postpetiole node measured in dorsal view, excluding helcium.

PPW Postpetiole width: maximum width of postpetiole node measured in dorsal view.

PTW Petiole width: maximum width of petiole node measured in dorsal view.

PW Pronotal width: maximum width of pronotum measured in dorsal view.

SL Scape length: maximum scape length excluding basal condyle and neck.

SPST Distance between center of propodeal stigma and spine tip. The stigma center refers to midpoint defined by outer cuticular ring but not to center of stigma opening, which may be positioned eccentrically.

WL Weber’s length: diagonal length of mesosoma in lateral view from postero-ventral margin of propodeal lobe to anterior-most point of pronotal slope, excluding the neck.

**Indices**.

CI Cephalic index: HW/HL × 100

DPeI Dorsal petiole index: PTW/PTL × 100

DPpI Dorsal postpetiole index: PPW/PPL × 100

LPpI Lateral postpetiole index: PPL/PPH × 100

OI Ocular index: EL/HW × 100

PeNI Petiolar node index: PTW/PW × 100

PPI Postpetiole index: PPW/PTW × 100

PpNI Postpetiolar node index: PPW/PW × 100

PSLI Propodeal spine index: SPST/HL × 100

SI Scape index: SL/HL × 100

### X-ray micro computed tomography and 3D images

The micro-CT scan was performed using a Zeiss Xradia 510 Versa 3D X-ray microscope operated with the Zeiss Scout-and-Scan Control System software (version 11.1.6411.17883). 3D reconstruction of the resulting scan raw data was done with the Zeiss Scout-and- Scan Control System Reconstructor (version 11.1.6411.17883) and saved in DICOM file format. Volume renderings, surface mesh generations, and virtual examinations were performed with Amira software (version 6.3.0). Post-processing of mesh data in order to generate a clean surface was done with Meshlab (version 1.3.3). The methodology for the virtual examination of 3D surface models, generation of 3D rotation videos, and virtual dissections follow Hita Garcia *et al*.^21^. For further details on micro-CT scanning and post-processing workflow pipeline, we refer to Hita Garcia *et al*.^21,23^.

## Data Availability

As in previous studies^21,23^, the specimens used in this study have been databased and the data are freely accessible on AntWeb (http://www.antweb.org). Each specimen can be traced by a unique specimen identifier attached to its pin. The Cybertype dataset has been archived and is freely available from the Dryad Digital Repository (23, 10.5061/dryad.4gg39k6). In addition to the cybertype data at Dryad, we also provide a freely accessible 3D surface model on Sketchfab (https://skfb.ly/6HYRz). All data needed to evaluate the conclusions in the paper are present in the paper. Additional data related to this paper may be requested from the authors.
